# Protective Antibody and CD8^+^ T-Cell Responses to the *Plasmodium falciparum* Circumsporozoite Protein Induced by a Nanoparticle Vaccine

**DOI:** 10.1371/journal.pone.0048304

**Published:** 2012-10-29

**Authors:** Stephen A. Kaba, Margaret E. McCoy, Tais A. P. F. Doll, Clara Brando, Qin Guo, Debleena Dasgupta, Yongkun Yang, Christian Mittelholzer, Roberta Spaccapelo, Andrea Crisanti, Peter Burkhard, David E. Lanar

**Affiliations:** 1 Malaria Vaccine Branch, Walter Reed Army Institute of Research, Silver Spring, Maryland, United States of America; 2 Institute of Materials Science, University of Connecticut, Storrs, Connecticut, United States of America; 3 M.E. Müller Institute for Structural Biology, University of Basel, Basel, Switzerland; 4 Department of Experimental Medicine, Microbiology Section, University of Perugia, Perugia, Italy; 5 Imperial College London, London, United Kingdom; 6 Department of Molecular and Cell Biology, University of Connecticut, Storrs, Connecticut, United States of America; Tulane University, United States of America

## Abstract

**Background:**

The worldwide burden of malaria remains a major public health problem due, in part, to the lack of an effective vaccine against the *Plasmodium falciparum* parasite. An effective vaccine will most likely require the induction of antigen specific CD8^+^ and CD4^+^ T-cells as well as long-lasting antibody responses all working in concert to eliminate the infection. We report here the effective modification of a self-assembling protein nanoparticle (SAPN) vaccine previously proven effective in control of a *P. berghei* infection in a rodent model to now present B- and T-cell epitopes of the human malaria parasite *P. falciparum* in a platform capable of being used in human subjects.

**Methodology/Principal Findings:**

To establish the basis for a SAPN-based vaccine, B- and CD8^+^ T-cell epitopes from the *P. falciparum* circumsporozoite protein (*Pf*CSP) and the universal CD4 T-helper epitope PADRE were engineered into a versatile small protein (∼125 amino acids) that self-assembles into a spherical nanoparticle repetitively displaying the selected epitopes. *P. falciparum* epitope specific immune responses were evaluated in mice using a transgenic *P. berghei* malaria parasite of mice expressing the human malaria full-length *P. falciparum* circumsporozoite protein (Tg-*Pb/Pf*CSP). We show that SAPN constructs, delivered in saline, can induce high-titer, long-lasting (1 year) protective antibody and poly-functional (IFNγ^+^, IL-2^+^) long-lived central memory CD8^+^ T-cells. Furthermore, we demonstrated that these Ab or CD8^+^ T–cells can independently provide sterile protection against a lethal challenge of the transgenic parasites.

**Conclusion:**

The SAPN construct induces long-lasting antibody and cellular immune responses to epitope specific sequences of the *P. falciparum* circumsporozoite protein (*Pf*CSP) and prevents infection in mice by a transgenic *P. berghei* parasite displaying the full length *Pf*CSP.

## Introduction

The worldwide burden of malaria remains a major public health problem due to the lack of an effective vaccine against *Plasmodium falciparum,* the causative agent of the deadliest human malaria [1]. There is no recombinant or viral based vaccine that induces long-lasting protective immune responses. Protection studies conducted using RTS,S as well as other available data, suggest that a robust antibody response coupled to a vigorous *Pf*CSP epitope specific CD8^+^ T-cell response will likely be needed for a highly effective pre-erythrocytic stage malaria vaccine [2]–[4]. But the induction of both of these arms of the immune system by a single vaccine against malaria has been difficult to achieve. Nanoparticle based vaccines have been shown to be effective in the induction of immune responses in animal models without the need for an adjuvant [5]–[10]. Most of these particles are based on polymers that encapsulate antigen or create a solid support to which protein antigens are chemically coupled and therefore do not necessarily have control over an ordered array presentation of epitopes.

We have previously reported using the basic properties of amino acids and the tenets of structural biology to design a short linear protein monomer that combines with identical monomers to form a self-assembling protein nanoparticle (SAPN) with predictable and controllable conformation and presentation of epitopes. Furthermore, we demonstrated these SAPN could elicited a CD4^+^ T-cell dependent, high titer, long-lived protective antibody response against a mouse malaria, *P. berghei*, sporozoite challenge [11] in mice. We report here modifications of that original SAPN design to make a vaccine that could be used in humans against the malaria parasite *P. falciparum.* This required change in the core or scaffold to eliminate sequences that might cross react with human proteins. We also included three previously identified CD8^+^ T-cell epitopes from the *P. falciparum* circumsporozoite protein (*Pf*CSP) as well as copies of the central *Pf*CSP repeat sequence of four amino acids NANP. Since the target of the induced immune responses are directed against the single sporozoite protein, *Pf*CSP, we choose to utilize a transgenic *P. berghei* parasite clone that normally infects rodents [12] to test the efficacy of the vaccines. These transgenic parasites express full length *Pf*CSP on the surface of the sporozoite stage and infect mice similar to wild type *P. berghei* sporozoites thus allowing us to directly test the functionality of immune responses, both antibody and cellular, made against the *P. falciparum* CSP. As control vaccine constructs we designed monomers that when assembled would have scaffolds identical to those of the *Pf*CSP-SAPNs but displaying on their surface the B-cell epitope repeat amino acid sequence of the *P. vivax* VK210 CSP [13].

## Results

### Expression of Monomer Protein and Refolding to Form a Nanoparticle

**Figure 1:**
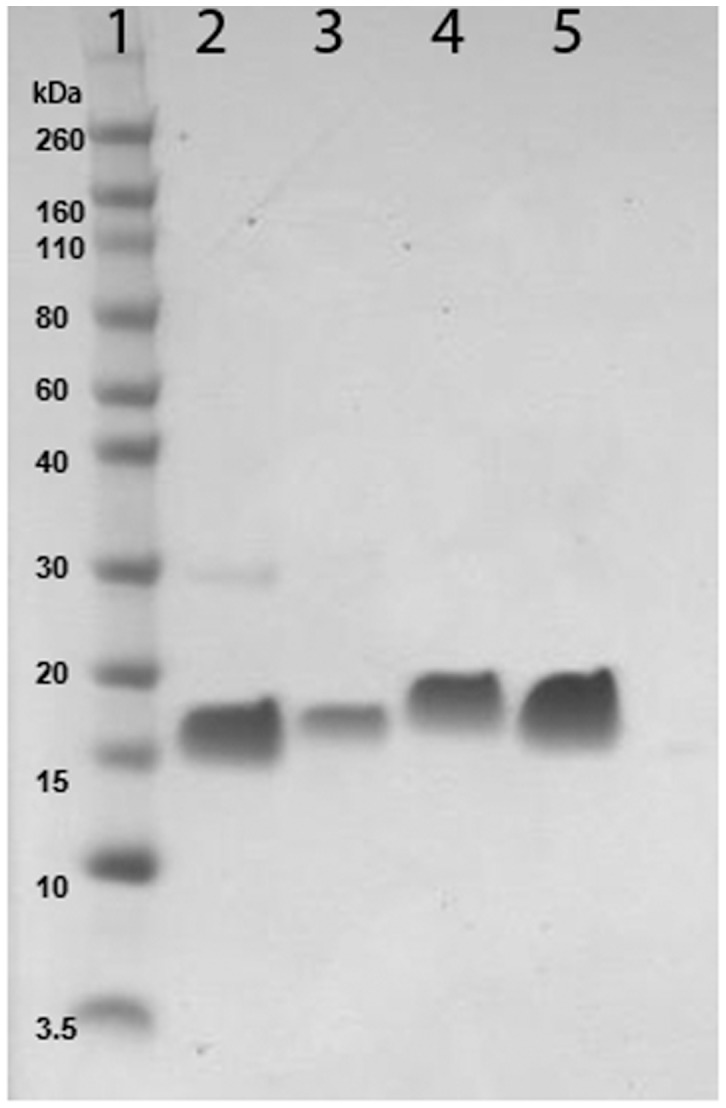
Analysis of purified monomers. (A) Coomassie Blue stained SDS-PAGE gel of monomer proteins. Lane 1: Molecular weight marker proteins; Lane 2: *Pf*CSP monomer; Lane 3: *Pv*CSP monomer; Lane 4: *Pf*CSP-KMY monomer; Lane 5: *Pv*CSP-KMY monomer.

**Figure 2:**
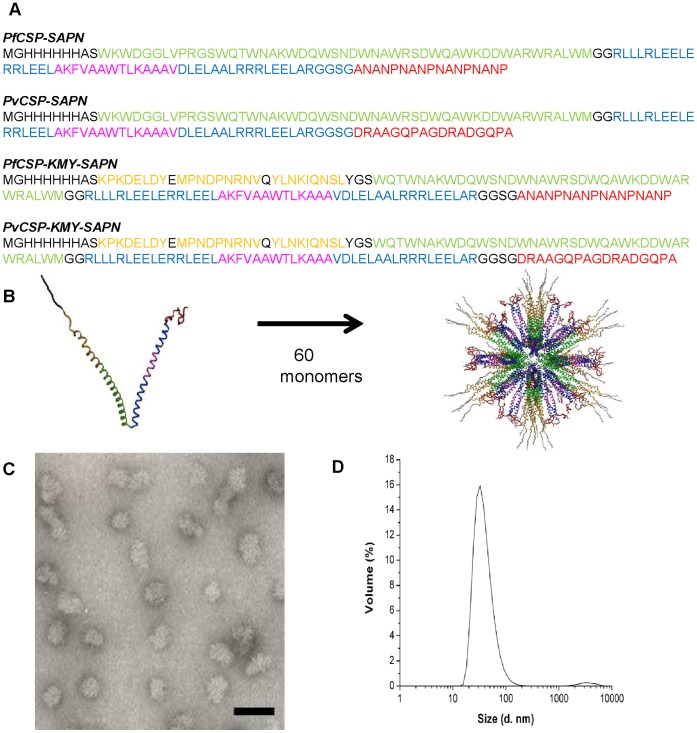
Sequences, formation and structural analysis of SAPN. (A) The amino acid sequences of the monomers used to make the SAPN in this study. Black: flanking regions (His-tag, thrombin cleavage site, proteosomal cleavage sites, linkers). Green: coiled-coil pentamer domain (Trp-zipper); Blue: coiled-coil trimer domain; Red: predicted B-cell epitopes of *P. falciparum* or *P. vivax* CSP repeat region; Yellow: predicted human HLA restricted CD8^+^ T-cell epitopes *P. falciparum* CSP; Magenta: universal CD4 T-helper epitope (PADRE) as a part of the trimer domain. (B) SAPNs are formed by the oligomerization of 3- and 5-stranded coiled-coiled domains within a single polypeptide monomer. Shown is the *in silico* prediction of the SAPN with icosahedral symmetry. Colors are representative of the sequences as described in (A). (C) Individual nanoparticles are visualized using transmission electron microscopy. The bar represents 100 nm. (D) The size distribution of the nanoparticles in solution is monitored using dynamic light scattering.

The gene for each monomer was cloned into a bacterial expression plasmid and transformed into *E. coli* cells for expression. Purity of the monomer was determined by SDS-PAGE (**Figure 1** **)**. After purification the denaturant was removed and self-assembly of each of the different monomers (**Figure 2A**) into nanoparticles was driven by the interaction of the trimeric and pentameric oligomerization domains creating α-helical rod-like coiled-coils [14] (**Figure 2B**). By both transmission electron microscopy and dynamic light scatter measurements the final SAPNs had a size of about 40 nm and formed uniform, non-aggregating particles (**Figure 2C, D**).

### SAPN Vaccines Expressing *P. falciparum* CSP (*Pf*CSP) Repeat Region B-cell Epitopes Protect Against a Transgenic *P. berghei* Sporozoites Displaying the *Pf*CSP

**Figure 3:**
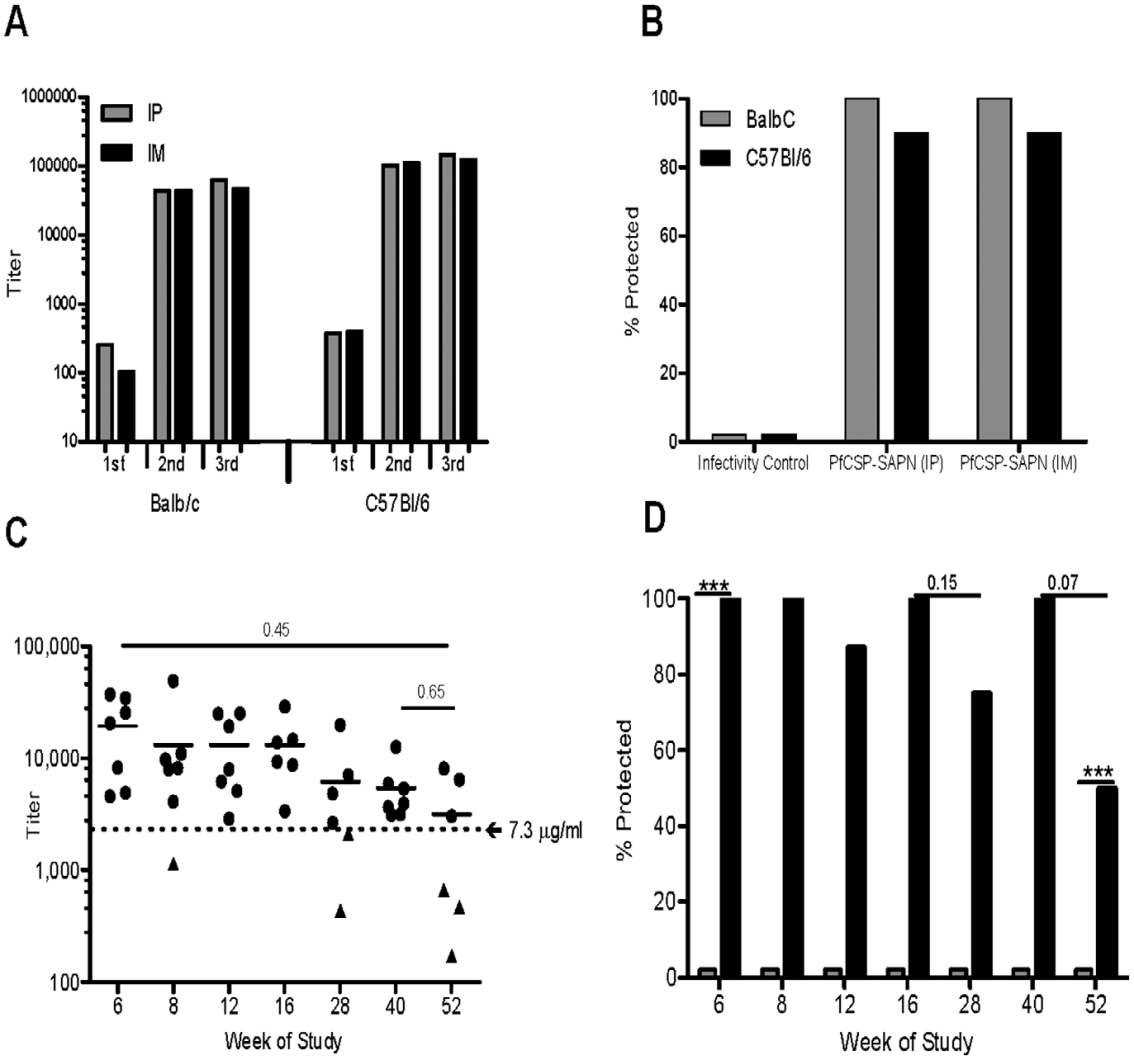
Antibody responses and protective efficacy induced by SAPN vaccinations in Mice. C57BL/6 and Balb/C mice make high titer antibodies when immunized with the *Pf*CSP SAPN. (A) Mice were given vaccine intraperitoneally (i.p.) or intramuscularly (i.m.) at wk 0, 2 and 4. Titers were determined 2 wks after each dose. n = 10 per group per experiment; data are representative of one of three independent experiments with similar results. (B) Two wks post 3^rd^ dose of *Pf*CSP-SAPN, C57BL/6 mice were challenged with 5,000 Tg-*Pb/Pf*CSP, Balb/C with 10,000, Tg-*Pb/Pf*CSP sporozoites. Shown are three separate experiments with C57BL/6 mice and two experiments with Balb/C mice. n = 10. Infectivity sham control mice were given PBS. Mice were considered protected if they had no detectable parasitemia by day 15 following challenge. (C) Titer of anti-*Pf*CSP repeat antibody of the serum from individual mice in each SAPN vaccinated group two days before challenge. The line represents the mean titers of the individual mice in that group. n = 6 or 7 depending if serum was available. Those SAPN immunized mice that were protected against malaria are represented by (•); those that developed parasitemia and died by (▴). (D) Protection following challenge. At the predetermined time point, from wk 6 to wk 52 of the study, C57BL/6 mice in a select immunized group (n = 6 or 7) and a time matched PBS-sham vaccinated group were challenged with 5,000 sporozoites. Mice receiving *Pf*CSP-SAPN immunization (black bars) and matched sham PBS vaccinated mice (white bars). ***P<0.0001.

Following two or three doses of vaccine high titer epitope specific antibodies to the NANP peptide were produced in both C57BL/6 and Balb/c mice (**Figure 3A**). Following challenge with sporozoites of the Tg-*Pb/Pf*CSP parasite two wks post third dose of vaccine 90% to 100% of mice were protected compared to 0% in the unimmunized infectivity control mice (**Figure 3B** **&** **Table 1**). Because our standard route of SAPN administration, i.p., is not a route commonly used to deliver vaccines to humans we tested i.m. administration in parallel experiments. The route of administration of *Pf*CSP-SAPN made no statistically significant difference in either the NANP-specific antibody titers observed (p = 0.45) (**Figure 3A**) or the levels of protection achieved (p = 0.99) in either strain of mice tested (**Figure 3B**).

**Table 1:** Consistency of protection against lethal challenge with malaria parasites in mice immunized with *Pf*CSP-SAPNs.

	% Protected (no. protected/no. immunized)			p-value	
Mouse Stain	Exp 1	Exp 2	Exp 3	Between Exp	To strain control
Balb/c	100(10/10)	100(10/10)	nd	0.99	<0.001
C57BL/6	100(10/10)	90(9/10)	90(9/10)	0.98	<0.001
Infectivity control Balb/c	0(0/10)	0(0/10)	0(0/10)	0.99	
Infectivity control C57BL/6	0(0/10)	0(0/10)	nd	0.99	

*Pf*CSP-SAPN vaccines were administrated at wk 0, 2 and 4 in three independent experiments Two wks post 3^rd^ dose of *Pf*CSP-SAPN, C57BL/6 mice were challenged with 5,000 and Balb/C with 10,000, Tg-*Pb/Pf*CSP sporozoites. Shown here are three separate experiments with C57BL/6 mice and two experiments with Balb/C mice. n = 10. Infectivity sham control mice were given PBS. Mice were considered protected if they had no detectable parasitemia by day 15 following challenge. nd = not determined.

### Protective Antibody Response is Long-lived

**Figure 4:**
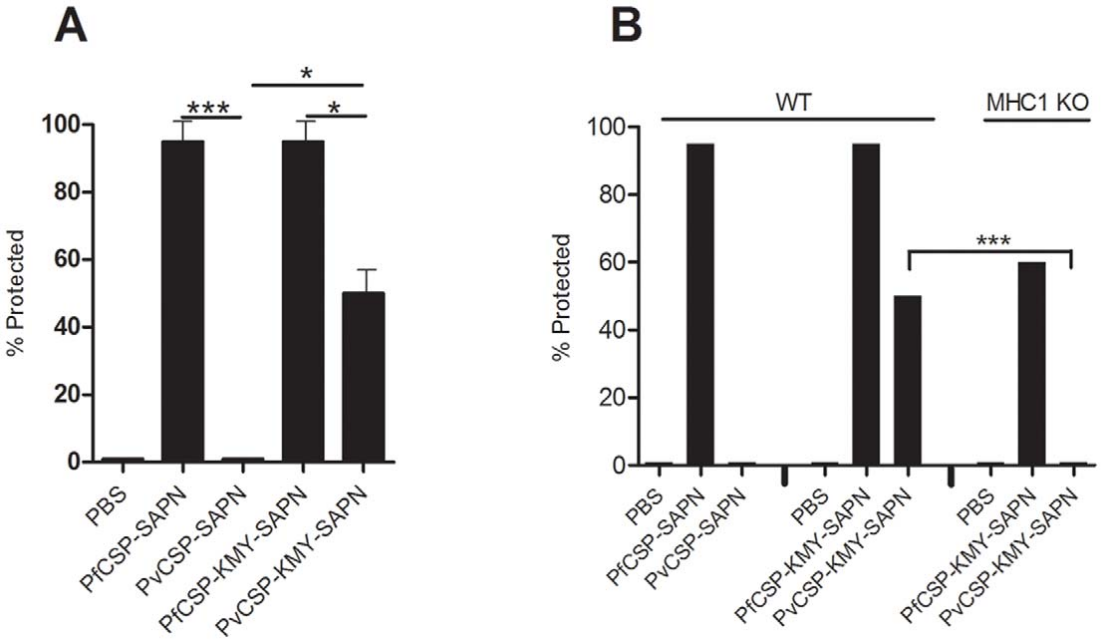
SAPN vaccination induces protective cellular immune responses in mice. SAPN based vaccines present CD8^+^ T-cell epitopes to stimulate a protective cellular immune response. (A) C57BL/6 mice immunized with a SAPN containing only *P. falciparum* CSP B-cell epitopes (*Pf*CSP-SAPN) or a SAPN containing both *P. falciparum* CSP B- and T-cell epitopes (*Pf*CSP-KMY-SAPN). n = 10; Error bars show means ± s.d. of three separate experiments. (B) Either sera or total splenocytes were transferred from immunized mice to naïve mice which were challenged 24 h post-transfer. n = 10; data shown is one of two experiments. (C) In order to determine if CD8^+^ T-cells were involved in protection we immunized wild-type C57BL/6 mice (WT) and MHC Class I knockout (MHC1 KO) mice with SAPN containing *Pf*CSP specific CD8^+^ epitopes. Mice were challenged with 5,000 Tg-*Pb/PfCSP* sporozoites. n = 10. *P<0.01; ***P<0.0001.

One of the most desirable qualities of a vaccine is long-term induction of an effective immune response. This would be especially advantageous for a malaria vaccine because in many areas where malaria is endemic there are periods of high or low transmission paralleling rainy and dry sessions, respectively. To investigate how long after immunization with *PfCSP-*SAPN protective antibody levels would persist, we immunized mice at wk 0, 2 and 4 and challenged them either at wk 6, 8, 12, 16, 28, 40 or 52 of the study with the Tg-*Pb/Pf*CSP sporozoites. After the third dose of vaccine the mice did not receive booster doses of vaccine nor were they exposed to parasites until the day of challenge. Mice maintained high antibody titers out to wk 52 (**Figure 3C**). Even up to wk 40, 75% to 100% of mice were protected following challenge and 50% of animals were protected at wk 52 (**Figure 3D**). One mouse from the wk 16, wk 28 and wk 52 test groups died before challenge from non-malaria, non-vaccine related causes. Analysis of pre-challenge antibody titers and avidity index of individual mice revealed that while there was no significant difference (p = 0.45) in the group mean pre-challenge antibody titers, those individual mice whose titer dropped below about 2200 (7.3 µg/ml) (**Figure 3C**) and avidity index ≤0.32 were not protected. All mice with anti-*Pf*CSP-SAPN antibody avidity index ≥0.46 were protected (**Table 2**). Whereas greater than 95% mice immunized with the *Pf*CSP-SAPN were protected from a lethal challenge none were protected if the animals were immunized with SAPN with an identical scaffold to the *Pf*CSP-SAPN but displaying *P. vivax* CSP repeat epitopes on its surface, the *Pv*CSP-SAPN (**Figure 4A**).

**Table 2:** Anti-SAPN antibody titer, avidity index and protection in mice immunized with *Pf*CSP-SAPNs.

Mouse ID	ELISA Ab Titer	Concentration of IgG (ug mL^−1^)	Avidity index of Ab	Status of protection
#590	3038	9.24	0.46	P
#591	466	6.00	0.32	NP
#592	668	1.75	0.24	NP
#895	6430	16.11	0.55	P
#979	8140	34.94	0.55	P
#980	171	0.09	0.22	NP

Analysis of sera from individual mice immunized with *Pf*CSP-SAPNs for anti-SAPN specific antibody titer, IgG concentration and avidity index vis-à-vis the protection status of each mouse. All sera used in this analysis were taken 48 wks post-3^rd^ immunization (wk 52 of study), one day before mice were challenged. P = protected from challenge dose of transgenic sporozoites. NP = not protected from challenge dose of transgenic sporozoites.

### SAPN Platform can Present T-cell Epitopes to Induce Protective CD8^+^ T-cells

**Figure 5:**
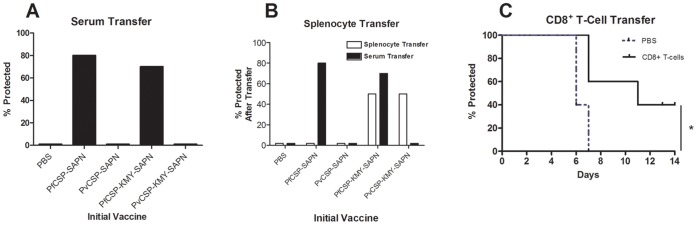
Sera or Cell Transfer Studies. (**A**) Pooled sera isolated from mice immunized with *Pf*CSP-SAPN or *Pf*CSP-KMY-SAPN but not sera from *Pv*CSP-SAPN or *Pv*CSP-KMY-SAPN immunized mice transferred to naïve mice conferred protection from challenge with Tg-*Pb/PfCSP* sporozoites. (**B**) In a separate experiment sera or washed splenocytes from mice were transferred. Whereas sera from *Pf*CSP-SAPN or *Pf*CSP-KMY-SAPN transferred protection sera from *Pv*CSP-SAPN or *Pv*CSP-KMY-SAPN immunized mice did not. On the contrary, total splenocytes from *Pf*CSP-KMY-SAPN or *Pv*CSP-KMY-SAPN transferred protection while splenocytes from *Pf*CSP-SAPN or *Pv*CSP-SAPN did not. (**C**) Two wks post final immunization with *Pf*CSP-KMY-SAPN 1.33×10^6^ enriched CD8^+^ T-cells were adoptively transferred to naïve animals which were then challenged 72 hrs post transfer. *P<0.05. Mice were challenged with 5,000 Tg-*Pb/PfCSP* sporozoites. n = 10.

Between 90% and 100% of mice immunized with *Pf*CSP-KMY-SAPN, and 40–60% of *Pv*CSP-KMY-SAPN-immunized mice were protected against lethal challenge (**Figure 4A**). While the protection in *Pf*CSP-KMY-SAPN immunized mice could be attributed to both antibody and cell mediated immune responses, the protection in *Pv*CSP-KMY-SAPN–immunized mice could only be attributed to a protective cellular response since antibodies to *P. vivax* CSP repeat epitopes do not cross-react with epitopes in *P. falciparum* CSP repeat region or against the Tg-*Pb/Pf*-CSP parasite. To further determine that cells and not antibodies were responsible for the observed protection in the experiments involving *Pv*CSP-KMY-SAPN we immunized a strain of MHC Class1 knockout mice [15] with the SAPN containing the CD8^+^ T-cell epitopes (**Figure 4B**). Knockout mice immunized with *Pv*CSP-KMY-SAPN were not protected from challenge, whereas wild type mice receiving *Pv*CSP-KMY-SAPN were protected. Naïve mice that received sera from *Pf*CSP-SAPN or *Pf*CSP-KMY-SAPN immunized mice survived challenge and mice that received splenocytes or enriched CD8 T-cells from *Pf*CSP-KMY-SAPN immunized mice were protected (**Figure 5**). But neither splenocytes nor serum from *Pv*CSP-SAPN-immunized mice conferred protection to naïve mice and mice that received only serum from *Pv*CSP-KMY-SAPN-immunized mice were not protected (**Figure 5B**). However, mice were protected if they received splenocytes from *Pv*CSP-KMY-SAPN-immunized mice (**Figure 5B**). Finally, the passive transfer of highly enriched CD8^+^ T-cells from the liver and spleens of mice immunized with *Pf*CSP-KMY-SAPN conferred protection against Tg-*Pb/Pf*CSP sporozoite challenge of naïve recipient mice (**Figure 5c**). Together, these experiments strongly indicated that a SAPN vaccine platform carrying *P. falciparum* CSP CD8^+^ T-cell epitopes was capable of inducing CD8^+^ T-cells that were directly involved with the protection against an otherwise lethal challenge of sporozoites.

**Figure 6:**
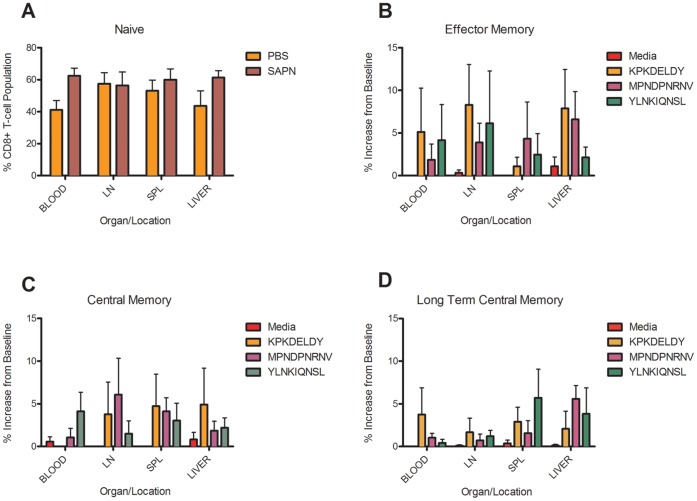
CD8^+^ T-lymphocyte population profiles. *Pf*CSP-KMY-SAPN immunized mice show induction of long-lived Ag-specific memory cells and residency in both secondary lymph and non-lymphoid organs (A–D). Two to five wks post final immunization total lymphocytes from designated organs were cultured, stimulated with peptides, stained and characterized by flow cytometry. Data are presented as a percentage of the CD8+ T-cell population normalized to peptide stimulated saline control minus media alone. Data was segregated based on phenotype and location for (A) naïve, (B) effector memory, (C) central memory, and (D) long-term central memory cell populations and characterized as Naïve, T_EM_, T_CM_ or T_LCM_ based on expression levels of CD44, CD62L, IL-2 and IFNγ. The FACS gating strategy is shown in Figure S1. Error bars represent the mean ± s.d. observed in 4 separate experiments, two mice each, over the course of 5 wks post vaccination. LN = lymph node; SPL = spleen.

An additional desirable quality of a malaria vaccine would be one that had the ability to induce multi-functional [16], long-term central memory CD8^+^ T-cells (T_LCM_) [17] that would accumulate at the sites of parasite replication [18], [19] and hopefully target infected cells for destruction. To determine if our SAPN vaccine induced T_LCM_ we investigated the phenotype of antigen-specific CD8^+^ T-cells following *Pf*CSP-KMY-SAPN immunization. We isolated and analyzed the lymphocytes from blood, draining lymph nodes, spleen and liver from individual mice for phenotypic markers of memory cells. We found that immunization did not disturb the homeostatic balance of the naïve T-cell compartment in the blood, spleen, liver or draining lymph nodes (**Figure 6A**). But we did observe the induction of about 2–8% increase over baseline of effector memory (CD44^hi^CD62L^lo^IFNγ^+^IL-2^+^), central memory (CD44^hi^CD62L^hi^IFNγ^-^IL-2^+^) or long-term central memory (CD44^hi^CD62L^hi^IFNγ^+^IL-2^+^) CD8^+^ T-cells in these organs (**Figure 6B–D** and **Figure S1A–E**). The long term central memory (T_LCM_) cells specifically responded by producing both IFNγ and IL-2 when exposed *in vitro* to each of the K, M, or Y peptides.

## Discussion

Our goal for this study was to determine if we could design and construct a SAPN that could be potentially used in humans to induce strong immune responses to the human malaria *P. falciparum* CSP epitopes. First, we demonstrated that SAPNs could elicit high-titer, high-avidity, and long-lasting protective antibodies to epitopes of the repeat region of the circumsporozoite surface protein of *P. falciparum*. Second, we established that SAPNs could induce antigen-specific CD8^+^ T-cells responses that played an active role in a protective immune response. These data demonstrate that unique, structure-designed nanoparticles, administered without adjuvant, have the ability to induce both antibody and cellular responses in mice that, together or independently, are able to sterilely protect mice against sporozoites which display on their surface the CSP of the human malaria parasite *P. falciparum*.

**Figure 7:**
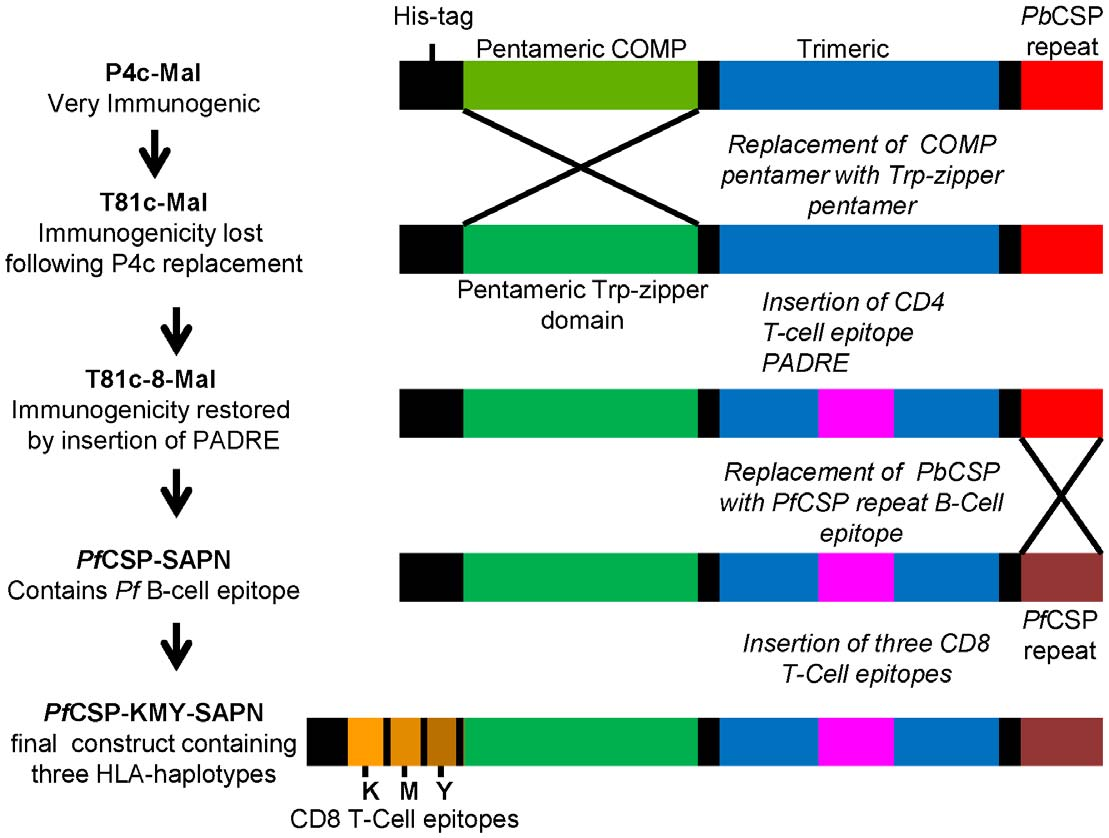
Schematic representation of redesigning the scaffold for SAPN-based *P. falciparum* CSP vaccine. The cartilage oligomerization matrix protein (COMP) was replaced with a *de novo* designed sequence (Trp-zipper) that, like COMP, forms a pentameric coiled-coil domain. Sequences coding for the universal CD4 T-cell helper epitope, the pan-allelic DR epitope (PADRE) were incorporated into the trimeric coiled-coil domain without disrupting the stoichiometry needed to oligomerize. The *P. berghei* circumsporozoite surface protein repeat (*Pb*CSP) epitopes were replaced with *Pf*CSP repeat epitopes to form *Pf*CSP-SAPN. Three *Pf*CSP CD8+ T cell epitopes were then engineered into the pentameric coiled coil domain to form *Pf*CSP-KMY-SAPN.

We had previously reported the use of a SAPN-based vaccine to induce protective immune responses in a mouse malaria model [11]. However, a part of the core scaffold (designated P4c) in our early mouse vaccine construct, P4c-Mal could not be used as basis for a vaccine destined to be used in humans because it contained sequences corresponding to the human cartilage oligomeric matrix protein (COMP). Therefore, to develop a SAPN-based vaccine that could be potentially used in humans, the COMP in P4c was replaced with a *de novo* designed high tryptophan content sequence (Trp-zipper) that, like COMP, formed a pentameric coiled-coil domain (**Figure 7**). Surprisingly, this new construct, T81c-Mal, did not induce antibody production in mice and protection against parasite challenge was lost. We reasoned that the removed COMP sequence contained a CD4 helper epitope and therefore we added the pan-allelic DR epitope (PADRE) [20] into the newly designed scaffold to make the construct T81c-8-Mal. This restored antibody production in mice and, subsequently, protection from challenge.

If sporozoites make their way to the liver they can avoid antibody by entering hepatocytes and undergoing developmental transformation and replication. In the liver stage of development CSP is no longer produced therefore all detectable CSP is a product of the initial invading sporozoite parasite [21]. The CSP is processed and peptide epitopes are presented on the hepatocyte surface in the context of MHC Class I molecules [22], [23]. It has been shown that *P. yoelii* CSP epitope specific CD8^+^ T-cells can kill hepatocytes containing developing malaria parasites [24]. But the induction of CSP-specific CD8^+^ T-cells using recombinant protein or a multiple antigenic peptide array vaccine has been difficult to achieve without the co-administration of a potent immuno-stimulators that are not suitable for human vaccine development [25]. Reports have indicated that CD8^+^ T-cell responses could also be induced by antigen-containing small particles (20–200 nm) that are trafficked to the lymph nodes where they are taken up by antigen presenting cells, processed and presented to adaptive immune cells [26]. To test if SAPN induced protective CD8^+^ T-cells we engineered on to the nanoparticle scaffold three predicted CD8^+^ T-cell epitopes (**K**PKDELDY, **M**PNDPNRNV & **Y**LNKQNSL) from the *P. falciparum* 3D7 clone. The epitopes were selected because of the prevalence of the human HLA B35, B7 and A2.1 haplotypes in the world’s population. About 40% of the world is HLA-A2 [27] with between 35–50% in every ethnicity in the USA [28]. In addition, the HLA-A2.1, HLA-B7 and HLA-B35 haplotype associated *Pf*CSP CD8^+^ T-cell epitopes have all been associated with protection from malaria in Africans and other ethnic groups [29]–[32] thus a vaccine based on these CD8^+^ T-cell epitopes should have a broad coverage throughout the world. If the vaccine is tested with a *P. falciparum* 3D7 clone challenge, all three CD8 epitopes will be present on the CSP of the invading sporozoite. However, of the three 3D7 clone epitopes chosen only one of the sequences, the M peptide, had a 100% homology match to the *Pf*CSP of the Wellcome strain CSP protein used in the transgenic parasite model (**Table 3**). This single 100% homology match, and perhaps the lower homology matched epitopes, did however induce protective CD8^+^ T-cells as demonstrated by the cell transfer studies presented in Figure 4 and Figure 5. This is encouraging because it suggests that a single vaccine containing several different HLA specific CD8^+^ T-cell epitopes could have broad coverage in a malaria endemic area with multiple parasite strains.

**Table 3:** Amino acid sequences of the three predicted *P. falciparum* CSP CD8^+^ T-cell epitopes (K, M and Y) based on binding to major human HLA haplotypes.

Peptide Name	K	M	Y
HLA Restriction	B35	B7	A2.1
Sequence in *Pf*- or *Pv*CSP-KMY-SAPN polypeptide	KPKDELDY	MPNDPNRNV	YLNKIQNSL
Sequence in 3D7 Strain CSP	KPKDELDY	MPNDPNRNV	YLNKIQNSL
Sequence in Wellcome Strain CSP	KPKDQLDY	MPNDPNRNV	YLKKIQNSL

Shown are comparisons to the known sequence in the 3D7 strain of *P. falciparum* CSP (used for human volunteer challenge trials) and to the Wellcome Strain of *P. falciparum* CSP sequence expressed in the Tg-*Pb/Pf*CSP sporozoites (used in these mouse studies). Underlined residues in Wellcome strain highlight the differences from the 3D7 strain.

One of the more interesting aspects of SAPN formulation is the ability to trigger immune responses without the need of an adjuvant. In our previous study we have shown that potential traces of LPS were not the reason for the adjuvant effect of SAPN [11]. Furthermore, *in vitro* studies have shown that SAPN activate dendritic cells in vitro to over-express the co-stimulatory molecules CD40 and CD86 (**Figure S2**). This is consistent with an adjuvant effect of SAPN. However, the exact mechanisms by which SAPN activate these dendritic cells as well as the receptor(s) involved in SAPN recognition by dendritic cells are still unclear and is currently under investigation.

The promise of vaccination against malaria depends on epitope selection and delivery to the immune system to induce strong, long-lasting immune responses. In this study, we performed the first essential steps to show the ability of a new vaccine platform to deliver *Pf*CSP B- and T-cell epitopes to induce long-lived humoral and cellular responses. Certainly the epitope approach would be of limited value if the selected sequences were highly variable. But, the central repeat region of the *Pf*CSP is highly conserved. While there is significant divergence in the CD8^+^ T-cell epitopes in the flanking regions we have shown that we can incorporate at least 3 different epitopes, encompassing major HLA haplotypes into the SAPN design which gives the vaccine a wider effective range. Using protein engineering we can change the vaccine to modify epitope presentation. For example, several different T-cell epitopes determined optimal for control of local parasite strains or improved responses from indigenous human MHC haplotype populations can be engineered into the monomer sequence to be assembled into the final vaccine product. The use of rodent transgenic parasites expressing *P. falciparum* molecules represented a powerful and convenient model to dissect and evaluate the effectiveness of the immune responses against a human malaria antigen. In future studies with human volunteers we will aim to demonstrate the safety and immunogenicity of this nanoparticle malaria vaccine and to test its effectiveness against a challenge of *P. falciparum* sporozoites.

## Materials and Methods

### Ethics Statement

Research with mice was conducted in compliance with the Animal Welfare Act and other federal statutes and regulations relating to animals and experiments involving animals and adheres to principles stated in the Guide for the Care and Use of Laboratory Animals, NRC Publication, 1996 edition. All animal work was conducted under protocols approved by the WRAIR/NMRC Institutional Animal Care and Use Committee.

### Redesign of the Scaffold

The redesign of the core scaffold to be use as the basis for human SAPN vaccine constructs was as schematically presented in **Figure 7**. The DNA sequence encoding the cartilage oligomerization matrix protein (COMP) was replaced with sequence that encodes a *de novo* designed Trp-zipper sequence: WQTWNAKWDQWSNDWNAWRSDWQAWKDDWARWRALWM, that forms a pentameric coiled-coil domain [33] (**Figure 2**). Sequences coding for the universal CD4 T-cell helper epitope, the pan-allelic DR epitope (PADRE) was incorporated into the trimeric coiled-coil domain without disrupting the stoichiometry needed to oligomerize followed by a replacement of the *P. berghei* circumsporozoite surface protein repeat (*Pb*CSP) epitopes with *Pf*CSP repeat epitopes to form *Pf*CSP-SAPN. Three *Pf*CSP CD8^+^ T-cell epitopes were then engineered on to the pentameric coiled coil domain to form *Pf*CSP-KMY-SAPN.

### *P. vivax* CSP Containing SAPN

Using the plasmids that expressed the *Pf*CSP-SAPN and *Pf*CSP-KMY-SAPN monomer proteins we switched the sequences that encoded the *Pf*CSP repeats for sequences that were designed to express monomer proteins containing the *P. vivax* CSP (VK210) repeat sequence DRAAGQPAGDRADGQPA (**Figure 2**).

### Plasmids, Protein Expression, Purification and Characterization

Plasmids (pET24 backbone) containing inserted genes to encode the desired monomer proteins were transformed into BL21 (DE3) *E. coli*, Tuner strain, cells and grown in LB Broth to OD_600 = _0.8 and induced with 0.5 mM IPTG for 2 h. Cells were collected by centrifugation and resuspended in 40 mL cold Cracking Buffer (6 M guanidine-HCl, 20 mM Tris-HCl, 100 mM NaH_2_PO_4_, pH 8.0) and disrupted by a single pass through a high-pressure microfluidizer (Model 1109; Microfluidic Corp.). The total cell lysate was centrifuged at 12,000 rpm, 30 min. The monomer protein was purified from the supernatant by nickel nitrilotriacetic acid (Ni-NTA)-agarose chelating resin affinity chromatography followed by two polishing ion-exchange chromatography steps utilizing an FPLC system (AKTA Purifier, GE Healthcare). The first, SP-Sepharose, enriched the monomer protein and the second, Q-Sepharose, bound and removed endotoxin. Throughout the purification protocol the monomer was kept in a denatured state by maintaining an 8 M urea concentration in the buffer. Buffer pH and salt concentrations were changed as required to retain or elute the monomer protein. All elutions were monitored by UV absorbance at OD_280_. The monomers were allowed to combine into a nanoparticle by dialysis of the final eluted protein solution against multiple changes of Refolding Buffer (20 mM Tris-HCl, 5% Glycerol (vol/vol), pH 7.5) to remove the urea. The nanoparticle size was determined by dynamic light scattering (DLS) and transmission electron microscopy (TEM) as described below. Endotoxin contamination of the final sample was determined by the pyrochrome *Limulus* amebocyte lysate (LAL) test (Associates of Cape Cod) following the manufacturer’s recommended instructions and was routinely <10 EU µg^−1^ of protein. Aliquoted vials of material were quick frozen on dry ice and stored at −80°C. For use a vial of sample was quickly thawed to RT and an aliquot was checked by DLS for confirmation of particle integrity.

### Transmission Electron Microscopy

The sample was negatively stained with 1% (wt/vol) uranyl acetate (SPI Supplies) and observed with a FEI Tecnai T12 S/TEM at an accelerating voltage of 80 kV (FEI). The protein concentration of the construct was about 0.05 mg mL^−1^.

### Dynamic Light Scattering

DLS experiments were carried out on a Zetasizer Nano S Instrument (Malvern), with a 633 nm He-Ne laser. All measurements were carried out at RT in the final refolding buffer containing 20 mM Tris-HCl, pH 7.5 and 5% glycerol (v/v).

### Mice

Six-to-eight-wk old female C57BL/6 (H-2^b^) and BALB/C (H-2^d^) mice were used for most protection experiments. In addition, an MHC Class I (MHC I) knockout (KO) strain (B6.129P2-*B2m^tm1Unc^*/J) was used to determine the role of CD8^+^ T-cells in the immune response to the SAPN. All mice were obtained from The Jackson Laboratory.

### Immunization

All mice were immunized either intramuscularly (i.m.) or intraperitoneally (i.p.) with 10 µg of protein in 0.1 mL of Refolding Buffer per immunization, on d 0, 14 and 28 unless otherwise noted.

### Challenge with Parasites

A clone of the transgenic *P. berghei* parasite [12], expressing the full-length *P. falciparum* CSP was used. In this transgenic parasite (Tg-*Pb/Pf*CSP) the endogenous *csp* gene from *P. berghei* had been replaced with the full-length *csp* gene from *P. falciparum* thus allowing testing of responses to both B- and T-cell *Pf*CSP specific epitopes. The sporozoite of this clone has been previously demonstrated to invade mouse hepatocytes and initiate a lethal blood infection in mice [12], [34]. Cryopreserved mouse blood containing infected RBCs of the original clone was injected into C57BL/6 mice and after 5 days whole blood was isolated and cryopreserved for future used. For infectious sporozoites an aliquot of frozen blood stage parasites was thawed and injected into mice. When gametocytes were observed to be above 3% of infected cells by stained slide examination *Anopheles stephensi* mosquitoes were allowed to feed on the mice. Sporozoites were isolated after 18–22 d. C57BL/6 mice were challenged by i.v. injection with 5,000 sporozoites/mouse; Balb/C mice were challenged with 10,000 sporozoites/mouse.

### Determination of Infection Following Parasite Challenge

Parasitemia was determined by examining Giemsa-stained thin smears prepared with blood from each mouse from 6–15 d post-challenge. Parasitemic animals were euthanized immediately following detection of blood stage parasites and an animal was considered fully protected if no parasites were detected by 15 d post challenge.

### Determination of Antibody Titers

Antibody responses against the *Pf*CSP peptide (NANP)_6_ or *Pv*CSP recombinant protein VMP001 (containing 9 copies of the *Pv*CSP VK210 repeat) [35] were measured by the enzyme-linked immunosorbent assay (ELISA) as previously described [11]. Briefly, 96-well microplates (Dynax) were coated with 100 ng of the synthetic *Pf*CSP peptide (Eurogentec North America) or 50 ng of the VMP001, per well, overnight at 4°C and then blocked for 1 h at 37°C with Blocker™ Casein in PBS (Thermo Scientific Inc). Plates were washed three times (PBS, 0.05% Tween20) and incubated for 1 h at 37°C with individual mouse sera in duplicate wells per serum sample. Plates were washed again and incubated for 1 h at RT with 1∶5,000 diluted (10× diluted solution of Blocker™ Casein in PBS) secondary anti-mouse immunoglobin (total IgG, IgM and IgA) labeled with horseradish peroxidase (Southern- Biotechnology Associates). Plates were washed and developed by adding 2, 2′-azino-di (3-ethylbenthiazoline sulfonate)-peroxidase (ABTS) substrate (Kirkegaard & Perry Laboratories) for 1 h at RT. The reaction was measured using a BioTek Synergy™ 4 Microplate reader by determining optical density at 405 nm (OD_405_). Antibody titers were determined as the dilution which gave an OD_405_ = 1 by the SoftMax Pro v5.2 ELISA Analysis Software (Molecular Devices LLC).

### Determination of Concentration of Antibody

Serum from immunized mice were diluted to 1∶500 and incubated for one hour with Luminex microspheres covalently coupled with the CS repeat peptide [(NANP)_7_]. After washing, microspheres were incubated for an additional hour with 100 µl of goat anti-mouse IgG-PE (Jackson ImmunoResearch). Samples were analyzed by Luminex® 200™ (Luminex Corporation, Austin, Tx) and the antibody concentration (µg/mL) interpolated from an 8-point standard curve prepared using dilutions of a monoclonal antibody with known concentration.

### Determination of anti-SAPN Antibody Avidity Index (AI)

The AI of anti-SAPN antibodies was determined as previously described [11]. Briefly, concentration of sodium isothiocynate (NaSCN) that eluted 50% of the total bound IgG was first determined. Then, two duplicate serial dilutions for a second ELISA were done (in duplicate wells) on a single plate. After three washes with PBS-T and one wash with PBS, 100 µL NaSCN (1.5 M, dissolved PBS) was added to one set of the serial dilutions on the plate and plain PBS to the other dilution series. After 15 min incubation, all wells were washed and developed as described above. The AI was calculated based on the ratio of the areas derived from under the curves obtained by the plot of optical density (OD_405 nm_) and log of the sera dilution in the ELISA experiment with and without NaSCN treatment.

### Adoptive Transfer of Serum or Cells

Serum or cells (total splenocytes or enriched CD8^+^ T-cells) were harvested two wks after the third immunization from mice for the adoptive transfer experiments. 100 µL of pooled serum (non-diluted) from immunized or non-immunized (injected with saline) mice was transfused to each of five 6–8 wk old naïve C57BL/6 female mice. Single cells suspensions were made from organs of immunized or saline control mice. CD8^+^ T-lymphocytes were enriched from the liver and spleens of immunized mice with a lineage depletion kit (the cocktail of antibodies was against CD4, CD11b, CD11c, CD19, CD45R (B220), CD49b (DX5), CD105, Anti-MHC Class II, and Anti-Ter-119 (Miltenyi Biotec)). Using antibodies to CD3e, CD4 and CD8 it was determined by flow cytometry that >98% of the enriched cells were CD8+ T-lymphocytes. A total of 20×10^6^ total splenocytes or 1.33×10^6^ enriched CD8^+^ T-cells were intravenously injected into naïve C57BL/6 mice. Mice were challenged with 5,000 sporozoites 24 h after receiving total splenocytes or 72 h after receiving enriched CD8^+^ T-cells.

### *In vitro* Cell Stimulation, Surface and Intracellular Staining

To assess cell profiles following *in vitro* stimulation with either KPKDELDY (K), MPNDPNRNV (M), or YLNKIQNSL (Y) peptides, single cell suspensions were made from the spleen, draining lymph nodes, blood and liver of PfCSP-KMY-SAPN immunized or saline control mice. Livers were perfused with saline, and lymphocytes were separated from hepatocytes using Percoll gradient treatment. Red blood cells from the spleen, liver and blood were lysed using an 8.3 g L^−1^ ammonium chloride solution. Cells were seeded in 96 well plates with 100 µg designated peptide or media alone for 5 h in the presence of GolgiStop. Cells were then harvested and stained for anti-mouse APC-conjugated anti-TCRβ (H57–597), Pacific Blue-conjugated anti-CD4 (RM4-5), FITC-conjugated anti-CD8α (53–6.7), PE-Cy5-conjugated anti-CD44 (IM7), PE-conjugated anti-CD62L (MEL-14), Alexa Fluor 700-conjuagted anti-IFN-γ (XMG1.2), and PE-Cy7-conjugated anti-IL-2 (JES6-5H4) all purchased from BD Pharmingen and analyzed by flow cytometry. Prior to intracellular staining with Alexa Fluor 700 anti-IFNγ (XMG1.2) and PE-Cy7-conjugated anti-IL-2 (JES6-5H4), cells were treated with Cytofix/Cytoperm reagents (BD Pharmingen) per manufacturer’s recommendations.

### Statistical Calculations

*p*-values between groups were calculated using unpaired students *t*-test. Evaluation of variance in (**Table 1**) was done by 1-way ANOVA. Comparison of Kaplan-Meier survival curves (**Figure 5C**) were done by log-rank (Mantel-Cox) test using Prism GraphPad v5.0.

## Supporting Information

Figure S1 **Representative gating strategy for determination of cell phenotypes.** (A) Cells from their respective organs were harvested from *PfCSP-KMY-SAPN* immunized or PBS sham immunized mice and were selected on a lymphocyte gate and (B) further characterized for expression of TCRβ. (C) TCR β+ cells were subdivided into CD8+ or CD4+ cells. (D) CD8+ T-cells were characterized as Naïve, TEM, TCM or TLCM based on expression levels of (E) CD44, CD62L, IL-2 and IFNγ. Shown is determination of TCM.
(TIF)

Figure S2 **In vitro re-stimulation of dendritic cells with SAPN induces expression of co-stimulatory molecules, CD40 and CD86.** Human dendritic cells were incubated overnight with media (negative control (Red)), 10 ng (Orange) or 1 ng (Green) LPS as positive controls or 5 µg test SAPN (Blue). Cells were then stained with markers of co-stimulatory molecules, CD40 (A) and CD86 (B).
(TIF)
