# Supplementary figures and images for: Protective Antibody and CD8+ T-Cell Responses to the Plasmodium falciparum Circumsporozoite Protein Induced by a Nanoparticle Vaccine
Source: PLoS One. 2012 Oct 29;7(10):e48304. doi: 10.1371/journal.pone.0048304 (PMC3483151; doi:10.1371/journal.pone.0048304)

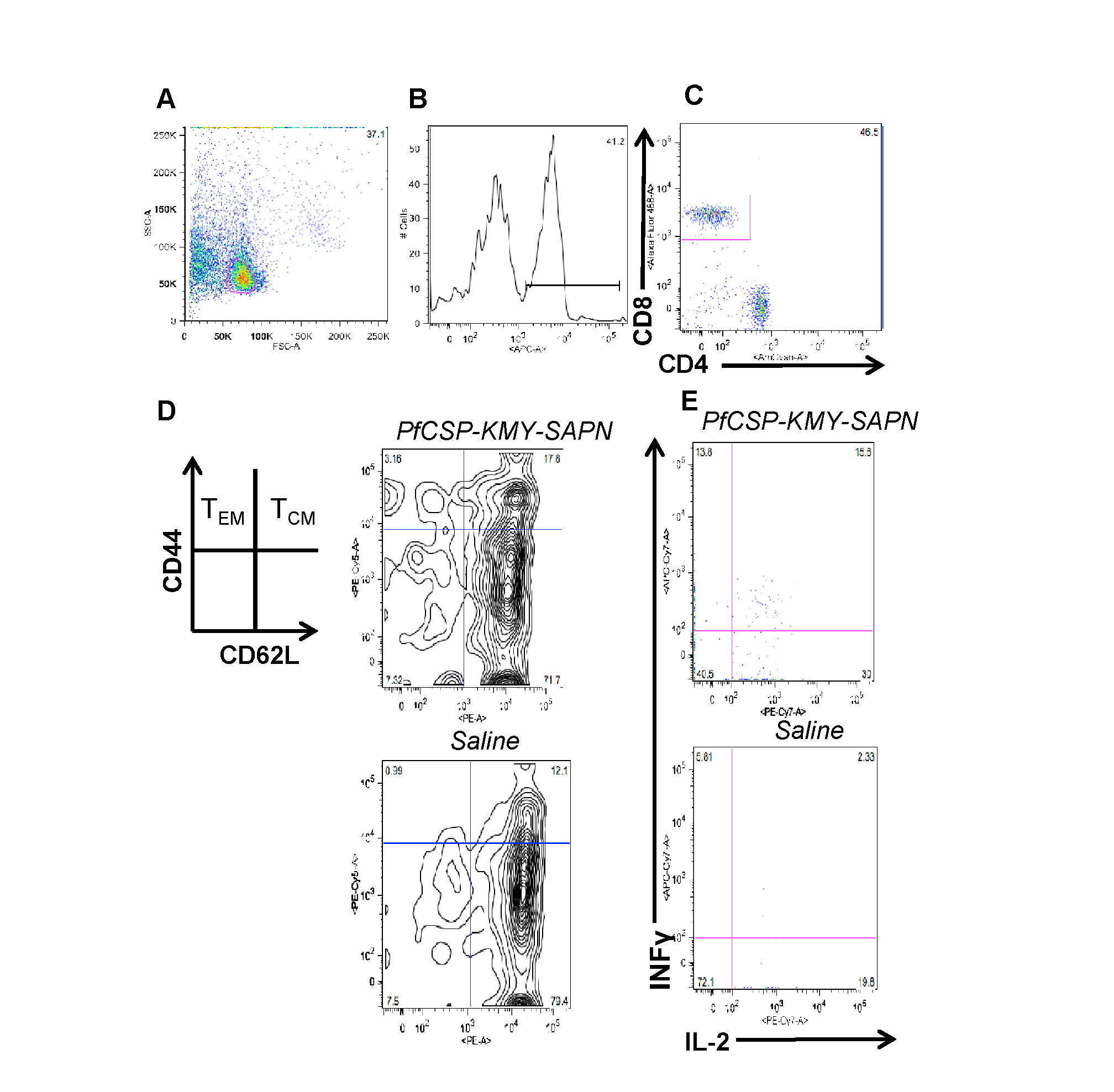

Supplement: Figure S1 — Representative gating strategy for determination of cell phenotypes. (A) Cells from their respective organs were harvested from PfCSP-KMY-SAPN immunized or PBS sham immunized mice and were selected on a lymphocyte gate and (B) further characterized for expression of TCRβ. (C) TCR β+ cells were subdivided into CD8+ or CD4+ cells. (D) CD8+ T-cells were characterized as Naïve, TEM, TCM or TLCM based on expression levels of (E) CD44, CD62L, IL-2 and IFNγ. Shown is determination of TCM. (TIF) [file pone.0048304.s001.tif]

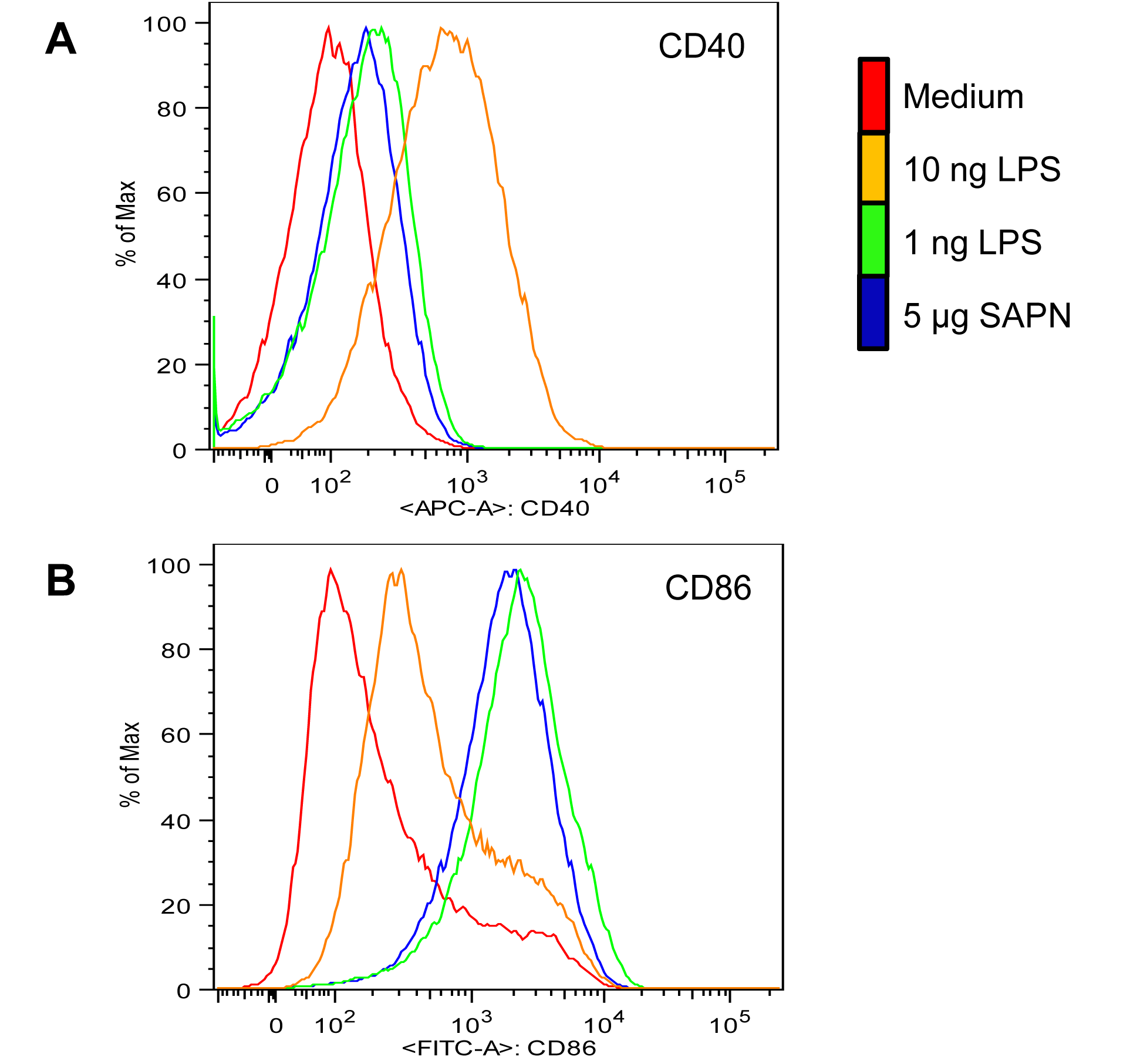

Supplement: Figure S2 — In vitro re-stimulation of dendritic cells with SAPN induces expression of co-stimulatory molecules, CD40 and CD86. Human dendritic cells were incubated overnight with media (negative control (Red)), 10 ng (Orange) or 1 ng (Green) LPS as positive controls or 5 µg test SAPN (Blue). Cells were then stained with markers of co-stimulatory molecules, CD40 (A) and CD86 (B). (TIF) [file pone.0048304.s002.tif]
